# Mediterranean Diet and Physical Activity Nudges versus Usual Care in Women with Rheumatoid Arthritis: Results from the MADEIRA Randomized Controlled Trial

**DOI:** 10.3390/nu15030676

**Published:** 2023-01-28

**Authors:** Panos Papandreou, Aristea Gioxari, Efstratia Daskalou, Maria G. Grammatikopoulou, Maria Skouroliakou, Dimitrios P. Bogdanos

**Affiliations:** 1 Department of Nutrition, IASO Hospital, 37 Chomatianou Str., Marousi, GR-15123 Athens, Greece; 2 Department of Nutritional Science and Dietetics, School of Health Sciences, University of the Peloponnese, GR-24100 Kalamata, Greece; 3 Department of Nutrition, General Hospital of Thessaloniki “G. Gennimatas”, 41 Ethnikis Aminis Str., GR-54635 Thessaloniki, Greece; 4 Unit of Immunonutrition and Clinical Nutrition, Department of Rheumatology and Clinical Immunology, Faculty of Medicine, School of Health Sciences, University of Thessaly, Biopolis, GR-41110 Larissa, Greece; 5 Department of Dietetics and Nutritional Science, School of Health Science and Education, Harokopio University, 70 Eleftheriou Venizelou Ave., GR-17676 Athens, Greece

**Keywords:** lifestyle medicine, immunonutrition, patient involvement, nutritional status, medical nutrition therapy, rheumatic disease, obesity, anti-inflammatory diet, 25(OH)D

## Abstract

In rheumatoid arthritis (RA), diet quality and nutritional status have been shown to impact the disease activity and adherence to the Mediterranean diet (MD) has been suggested as an anti-inflammatory regime to improve disease status and reduce cardiovascular risk. The Mediterranean DiEt In Rheumatoid Arthritis (MADEIRA) was a single-blind (statistician), two-arm randomized clinical trial, investigating the effects of a 12-week lifestyle intervention, including a personalized isocaloric MD plan with the promotion of physical activity (PA), supported through a clinical decision support systems (CDSS) platform, versus usual care in women with RA. Forty adult women with RA on remission were randomly allocated (1:1 ratio) to either the intervention or the control arm. The intervention group received personalized MD plans and lifestyle consultation on improving PA levels, whereas the controls were given generic dietary and PA advice, based on the National Dietary Guidelines. The primary outcome was that the difference in the MD adherence and secondary outcomes included change in disease activity (DAS28), anthropometric indices (BodPod), dietary intake, PA, vitamin D concentrations, and blood lipid profiles after 12 weeks from the initiation of the trial. At 3 months post-baseline, participants in the MD arm exhibited greater adherence to the MD compared with the controls (*p* < 0.001), lower DAS28 (*p* < 0.001), favorable improvements in dietary intake (*p* = 0.001), PA (*p* = 0.002), body weight and body composition (*p* < 0.001), blood glucose (*p* = 0.005), and serum 1,25(OH)_2_D concentrations (*p* < 0.001). The delivery of the MD and PA promotion through CDSS nudges in women with RA in an intensive manner improves the MD adherence and is associated with beneficial results regarding disease activity and cardiometabolic-related outcomes, compared with the usual care.

## 1. Introduction

Rheumatoid arthritis (RA) is one of the most common autoimmune diseases, inducing a systematic inflammatory state of the joints, gradually leading to bone resorption and cartilage erosion and the destruction of the affected joints [1,2]. Major symptoms include swelling of the joints, stiffness and pain, which negatively affect patients’ quality of life (QoL) [3]. Recent estimates of the prevalence of RA suggest a 1–2% global prevalence, with approximately two to three times more women being affected compared to the men [4,5]. In general, female patients tend to demonstrate worse outcomes than males. The treatment of RA aims in improving symptomatology, and reducing complications and lowering disease activity, allowing for the induction and sustainment of disease remission [3,6].

A plethora of genetic and environmental factors have been implicated in the pathogenesis of RA, with diet quality playing a significant role in the development of the entity, its pathophysiology, the manifestation of symptoms, and the management of RA [7,8,9]. Various dietary patterns, including the Mediterranean diet (MD), and individual nutrients including certain types of fatty acids and vitamin D, have been investigated for their potential associations with the development and prognosis of RA [10,11,12,13,14]. Dietary factors may interfere with several inflammatory pathways, influencing key pathophysiology features in RA [15]. For instance, a high-fat Western-type diet based on animal origin foods, which induces excess body weight and body fat, has been positively associated with the early onset of RA and a burdensome disease state [16,17]. A putative mechanism is the potential crosstalk between chronic obesity and RA inflammation, resulting in the upregulation of pro-inflammatory mediators, such as the tumor necrosis factor-α (TNF-α), interleukin-6 (IL-6), and C-reactive protein (CRP) [18]. Furthermore, Western-type diets decrease the odds of achieving and sustaining disease remission, inducing greater disease activity scores [19].

The MD is a predominantly plant-based diet that includes a variety of fruits, vegetables, whole grains, seeds, legumes, olive oils, and fish, characterized by a moderate intake of dairy products and a low consumption of animal fats [20]. Adherence to the MD and its individual components entails a variety of anti-inflammatory effects, alleviating RA symptomatology [10,11,21,22,23]. In parallel, the MD has been shown to exert protective and beneficial effects against obesity and cardiovascular (CV) and metabolic disorders [24,25,26].

The risk for the development of cardiovascular diseases (CVD) is markedly increased in RA as a result of the chronic inflammation in various body systems, including the heart and the vascular system [27,28]. In particular, the risk of developing heart failure is double in RA compared with the unaffected population, with patients demonstrating a preserved ejection fraction >50%, without necessarily any clinical evidence of coronary artery disease (CAD) [28]. For this, the European League Against Rheumatism (EULAR) recommends the multiplication of traditional cumulative CV risk scores in RA by ×1.5 in order to reflect the increased CVD risk associated with RA. In RA, accumulative CV risk factors are associated with poorer outcomes [29] and reduced survival [30]. Recently, the EULAR recommended lifestyle modifications, including adherence to MD, regular exercise and smoking cessation, as protective measures against the development and progression of CVD [31].

With both RA and CVD being predominantly a “female challenge” [4,5], the need to address the efficacy of lifestyle modifications, including the MD in women with RA, becomes urgent. In this manner, the present randomized controlled trial (RCT) aimed to evaluate the effect of a personalized MD plan delivered through a clinical decisions support system (CDSS) platform versus usual care, in women with an RA diagnosis.

## 2. Materials and Methods

### 2.1. Study Design and Protocol

The Mediterranean DiEt In Rheumatoid Arthritis (MADEIRA) was a single-blind (statistician), two-arm randomized controlled trial, investigating the effects of a 12-week personalized lifestyle intervention including MD and PA promotion, versus usual care in women with RA. The study’s protocol was registered at the Center for Open Science Framework (OSF, https://www.shorturl.at/krW24, accessed on 22 January 2023). The reporting of the study follows the consolidated standards of reporting trials (CONSORT) statement [32].

### 2.2. Ethical Clearance

The Ethics Committee of Iaso Hospital (Athens, Greece) reviewed and approved the protocol of the study (Approval Code #d310519). All principles of the Helsinki Declaration and its later amendments were adhered to, and the terms of Good Clinical Practice were applied. Informed consent was obtained from all subjects involved in the study.

### 2.3. Participants

Adult women with a definite RA diagnosis based on the American College of Rheumatology (ACR)/EULAR 2010 classification criteria [33] were recruited from the outpatient clinic of Iaso Hospital (Athens, Greece) during 2021–2. Information on the study aim, protocol, and methodology were provided to all patients through a detailed leaflet before recruitment. Patients were advised to read the leaflet in detail and sign the informed consent in two copies, keeping one. The study took place from November 2021 until April 2022, at Iaso Hospital, in Athens. The inclusion and exclusion criteria for participation in the study are detailed in Table 1.

### 2.4. Randomization Procedure

Patients were randomly allocated to the intervention or the control arm, on blocks of 1, using an online random allocation software (https://stattrek.com/, accessed on 28 January 2023). Investigators and patients were aware of the allocation. An independent researcher was responsible for the randomization. The statistician was blinded to the allocation (single blind).

### 2.5. Procedures and Tools

#### 2.5.1. Medical History and RA Specificities

A detailed medical history that included general information and disease-specific data (disease activity, symptoms, complications, treatment) was obtained by the attending rheumatologist specialist.

All women underwent clinical evaluation of RA through an objective physical articular examination, which was also used to calculate the Disease Activity Score of 28 joints (DAS28) [34] by the rheumatologist. The DAS28 combines data regarding the number of swollen and tender joints (based on 28 joints in total), perceived general health, and the acute phase response to the disease [34]. The score ranges between 0 to 9.4, with values exceeding 5.1 being indicative of an active disease and scores < 3.2 pointing to an inactive, well-controlled disease status [34].

#### 2.5.2. Dietary Intake and MD Adherence

A semi-quantitative Food Frequency Questionnaire (FFQ), previously validated in Greek patients with CVD, was employed for the assessment of the usual dietary intake of participants [35]. Portion sizes estimations were facilitated with the demonstration of food replicas and real-size food photos.

The Diet Analysis Plus (version 6.1, Wadsworth 2003) software was used to analyze individual FFQs and previous day 24 h diet recalls (treatment adherence). The daily dietary intake of saturated fatty acids (SFA), monounsaturated fatty acids (MUFA), total fat, dietary fiber, and vitamin D were calculated for each participant.

Adherence to the MD was assessed using the Mediterranean Diet Score (MedDietScore) [36]. The score is based on the reported frequency of consumption of 11 food groups, which are either representative, or not, of the MD pattern [36]. The total score is calculated by summing the scores of each individual food group and ranges between 0–55 in a linear manner, with greater scores being indicative of increased adherence to the MD and a lower risk for the development of CVD [36].

#### 2.5.3. Physical Activity (PA) Levels

The Greek version of the International Physical Activity Questionnaire (IPAQ) [37,38] for young and middle-aged adults was completed by all participants. The IPAQ measures health-related physical activity (PA) [38]. The IPAQ consists of 5 domains, each recording distinct activities, including (i) work-related PA; (ii) transportation PA; (iii) housework, house maintenance and caring for the family PA; (iv) recreation, sport and leisure PA; and (v) time spent sitting. The PA levels of each participant were expressed as metabolic equivalents of task (METs), in min per week.

#### 2.5.4. Anthropometric Indices

Body weight (BW) was measured in kg, to the nearest g, using the Air Displacement Plethysmography method (Bodpod^®^ Body Composition Tracking Systems, Life Measurement, Inc., Rome, Italy). Fat mass (FM) and fat-free mass (FFM) of participants were also estimated using the same device. For the Bodpod tests, participants did not perform any exercise or consume any foods and drinks for at least 2 h prior to the assessment, and were dressed in their underwear only [39], according to manufacturer guidelines.

Height was measured with a calibrated stadiometer to the nearest 0.1 cm (Seca 217, Seca, Hamburg, Germany).

Body mass index (BMI) of each participant was calculated as the ratio of BW (kg) to the square of height (m^2^). The BMI cutoffs for the classification of underweight (< 18.5 kg/m^2^), overweight (25 kg/m^2^ ≤ BMI < 30 kg/m^2^), and obesity (BMI ≥ 30 kg/m^2^) suggested by the World Health Organization (WHO) were applied [40] for the assessment of the BW status of participants.

#### 2.5.5. Blood Samples: Collection and Assays

Each patient provided morning whole blood samples (20 mL) for the isolation of the serum and plasma after an overnight fast. Ethylenediamine tetra-acetic acid (EDTA) was used for the plasma isolation. For the isolation of serum, whole blood was previously allowed to clot at room temperature for 20 min. Whole blood samples were centrifuged at 3000 rpm for 10 min at 4 °C. For all assays, freshly drawn blood samples were used.

Serum glucose (Glu), total cholesterol (TC), high-density lipoprotein (HDL), low-density lipoprotein (LDL), triglycerides (TG), and CRP were quantified with an automatic biochemical analyser using manufacturer’s reagents (Cobas 8000 modular analyser, Roche Diagnostics GmbH, Mannheim, Germany).

An automated chemiluminescence system (Cobase 801 analytical module, Roche Diagnostics GmbH, Mannheim, Germany) was used to determine the concentrations of 1,25-dihydroxyvitamin D [1,25(OH)_2_D] in the serum of the participants, using manufacturer’s reagents.

### 2.6. Intervention and Comparator

#### 2.6.1. Intervention

In the intervention arm, isocaloric personalized dietary plans according to the principles of the MD, patients’ energy expenditure requirements (EER), and food preferences were provided to the participants. In parallel, advice was also provided on increasing the PA levels according to the EULAR recommendations [31].

A CDSS platform was used for the delivery of the patient education, as previously detailed [41,42]. The specific CDSS was developed during 2016 and has been used in clinical practice as a complementary tool for the hospital’s dieticians and as a patient nudge tool [41,43]. CDSS platforms are employed to improve the delivery of healthcare interventions by facilitating medical decisions through the provision of targeted knowledge and health information [44]. Personalized login usernames and passwords were administered to all participating patients in the intervention arm, in order to have access to their personal CDSS database account, by which they could track their progress regarding BW, healthy eating, and PA levels.

The intervention lasted for a total of 12 weeks. During this time, two experienced registered dietitians (RDNs) communicated with participants on a biweekly basis through telephone calls to catch up, resolve possible queries, and provide support (A.G. and E.D.)

#### 2.6.2. Comparator (Control Arm)

Participants in the control arm did not receive a personalized MD plan, nor did they have access to the CDSS platform. In contrast, they were given generic dietary advice and PA recommendations that were in line with the Greek National Dietary Guidelines for adults [45].

#### 2.6.3. Treatment Adherence

According to the RDN’s instructions, all patients kept self-filled weekly food diaries that were evaluated remotely through CDSS (intervention group) or emails (control group). Unexpected phone calls were also made to participants to obtain 24 h diet recalls.

#### 2.6.4. Study Timepoints

All measurements were performed at the baseline and at the end of the intervention period, at 12 weeks.

### 2.7. Primary and Secondary Outcomes 

The primary outcome of the study was the difference (Δ) in the degree of adherence to the MD, assessed through the MedDietScore.

Secondary outcomes included differences (Δ) in disease activity (DAS28), dietary intake (SFA, MUFA, total fat, dietary fiber), as well as CV risk factors and inflammatory markers (BMI, FM, FFM, TC, HDL, LDL, TG, CRP levels), total PA levels, and 1,25(OH)_2_D concentrations.

### 2.8. Sample Size Calculation

A minimum sample size of 34 patients (17 per group) was deemed sufficient to result in a clinically important difference of 3.0 in the MedDietScore [standard deviation of mean (SD) = 3] using a two-tailed *t*-test with 80% power and a 5% level of significance (α).

### 2.9. Statistical Analyses

The Kolmogorov–Smirnov test was used to assess the normality of the distribution. Descriptive statistics were calculated for all parameters. Continuous data were expressed as mean ± standard deviation (SD), and dichotomous variables as counts (*n*) and proportions (%). 

Differences between the two arms were assessed using Student’s *t*-test for normally distributed variables or the Mann–Whitney U test for those not normally distributed. For investigating possible intra-group differences, a paired samples *t*-test or the Wilcoxon test was applied. Correlations between the MedDietScore and dietary, anthropometric, and biochemical parameters were assessed using either the Pearson’s or the Spearman correlation coefficients for normally distributed and not normally distributed variables, respectively. All correlations were assessed at the end of treatment.

Statistical significance was set at *p*-value < 0.05. All analyses were performed with the Statistical Package for the Social Sciences (SPSS) (version 21.0, SPSS, Inc, ΙΒΜ, Chicago, IL, USA) and the Jamovi project (version 1.2.27.0) [46].

## 3. Results

### 3.1. Participants

**Figure 1:**
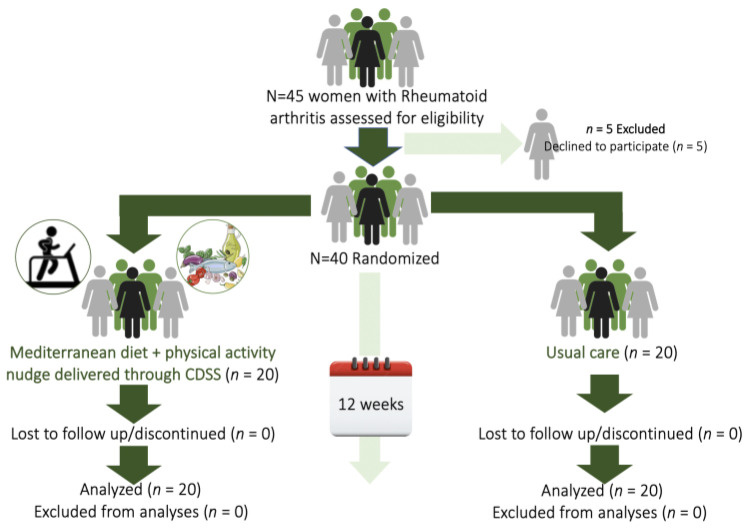
CONSORT [32] diagram of the study’s procedures.

The study sample comprised 40 women with RA meeting the inclusion criteria. All participants completed the trial and were incorporated in the final analyses, without any dropouts or patients lost-to-follow-up being recorded. The CONSORT diagram [32] of the study’s procedure is presented in Figure 1. All women inhabited the Attica region in Greece, were non-smokers, and reported consuming alcohol rarely or abstaining from alcohol.

Table 2 details the characteristics of the participants at the baseline. No differences were observed between the intervention and the control arm at the baseline regarding the participant age, anthropometric indices, body composition, dietary intake, blood markers, disease activity score, PA METs, and MedDietScore.

### 3.2. Mediterranean Diet Adherence and Dietary Intake

Table 3 presents the characteristics of the participants in each arm and pre-post intervention comparisons. Post-intervention, a significant increment was observed in the MD adherence among the participants in the intervention arm (*p* < 0.001), with a mean statistically significant increase of 3.75 units, while no change was observed among participants allocated to the control arm.

### 3.3. Disease Activity

DAS28 was significantly reduced at 3 months compared to the baseline among patients who received the intensive lifestyle intervention (*p* < 0.001). DAS28 correlated positively with the BMI (r = 0.330, *p* = 0.038) and dietary fat intake (r = 0.476, *p* = 0.002), and negatively with the MedDietScore (r = −0.452, *p* = 0.003).

### 3.4. Anthropometrics and PA Levels

At 12 weeks post-baseline, patients allocated in the intervention group exhibited a greater reduction in BW, BMI, and FM (*p* < 0.001) compared with the controls. PA levels were also improved in the intervention arm compared to the controls after 3 months (*p* = 0.002).

### 3.5. Dietary Intake

The post-intervention dietary intake of total fat, dietary cholesterol, and SFA were significantly lower in the intervention arm compared with the controls, while the consumption of MUFA and fiber were higher in the first compared with the latter (*p* ≤ 0.001). The MedDietScore correlated positively with the MUFA (r = 0.315, *p* = 0.047) and fiber intake (r = 0.477, *p* = 0.02), and negatively with the consumption of total fat (r = −0471, *p* = 0.002) and dietary cholesterol (r = −0.707, *p* = 0.001).

### 3.6. Blood Glucose, Serum Lipids, CRP and Vitamin D Concentrations

Both study groups exhibited greater serum vitamin D concentrations at 3 months after the initiation of the trial (*p* = 0.001), but, within the intervention arm, the mean increment was greater compared with that observed in the controls (*p* < 0.001). Serum vitamin D levels were negatively related to BW (r = −0.533, *p* < 0.001), FM as a percentage of BW (r = −0.363, *p* = 0.021), BMI (r = −0.560, *p* < 0.001), and the dietary intake of SFA (r = −0.348, *p* = 0.028). Vitamin D levels also correlated positively with the intake of MUFA (r = 0.436, *p* = 0.005) and dietary fiber (r = 0.510, *p* = 0.001).

No differences were noted with regard to the CRP and blood lipids levels between the intervention and comparator arms. On the other hand, blood glucose concentrations were improved among participants receiving the lifestyle intervention (*p* = 0.005).

Serum TG concentrations correlated negatively with the MedDietScore (r = −0.326, *p* = 0.004).

## 4. Discussion

The present study showed that a 12-week personalized MD plan, paired with PA promotion and delivered with the support of CDSS was successful in improving adherence to the MD, disease activity, PA levels, and a plethora of cardiometabolic outcomes among female patients with RA.

Adherence to the MD can be a useful tool to combat immune-mediated inflammatory diseases, including RA [23]. Results from other countries have shown a suboptimal adherence to the MD among patients with an RA diagnosis [47], with most patients adopting diets of poor quality in general, failing to meet the daily requirements for many nutrients [48,49,50,51]. A recent observational study conducted in Greece, suggested that female patients with RA demonstrated low-to-moderate adherence to the MD, with a mean MedDietScore equal to 29.55 [22]. In agreement with this, participants herein exhibited a mean baseline MedDietScore of 38.25, indicative of a moderate MD adherence and the adoption of a diet of mediocre quality. Among patients allocated to the intervention arm, adherence to the MD was further improved after 12 weeks of the intervention. In parallel, previous research has shown that adherence to the MD is associated with improved health perception regarding RA and general health [52]. Previous studies on the MD pattern have also shown improvements in the subjective measures of RA disease activity [28,29,30].

RCTs delivering traditional Cretan MD or other anti-inflammatory dietary interventions in patients with RA have revealed improvements regarding disease activity [53,54,55], whereas others suggested differences in the level of MD adherence between patients with low and high disease activity [22], suggesting that diet quality and composition might affect RA status. In the current trial, improvement in MD adherence was associated with ameliorated disease status, as evidenced by lower disease activity (DAS28 was negatively associated with MD adherence). This might have been the result of ameliorated dietary intake, with the observed improvements in the consumption of MUFA and fiber and the reduction in SFA intake being possible precipitating factors. The anti-inflammatory effects of MUFA in RA have been well-discussed in the context of the MD. In the TOMMOROW cohort [56], the dietary intake of MUFA was lower among patients with RA compared with healthy controls, while the ratio of the MUFA/SFA intake was positively associated with RA remission. The MD is considered as the ultimate anti-inflammatory dietary pattern, as it focuses on eating whole, plant-based foods that are rich in fiber and phytonutrients, while maintaining a stable glycemic response [57]. Dietary fiber is not digested in the small intestine, but upon fermentation by the colonic microflora, several microbial metabolites are produced that possess health-promoting effects. For instance, short chain fatty acids have been associated with improvements regarding microbial dysbiosis and the regulation of inflammatory biomarkers, such as plasma CRP, TNF-α, and IL-6, which are potent triggers of the RA disease activity [58]. There is evidence that a low-fiber dietary pattern may be linked to increased RA disease activity. For example, Elahi and associates showed that patients with RA on a low-fiber diet exhibited greater disease activity compared with those adhering to diets of higher-fiber contents [59].

In the present study, disease activity was also associated with BMI. The overall combined prevalence of overweight and obesity in the sample was high, namely 35% and 10%, respectively, and this observation is in accordance with previous studies indicating that excessive body weight is an important comorbidity in RA [22]. Patients with RA and comorbid obesity have been shown to exhibit greater DAS28 compared with the normoweight or overweight patients [60]. In RA, overweight and obesity consist of common results of long-term corticosteroid use and physical inactivity [61]. According to Padel, however [62], despite the seemingly worse disease activity, when imaging techniques are applied, patients with RA and obesity appear to have less inflammation and reduced rates of radiographic progression through time. A better diet quality in terms of adopting the MD pattern could be the possible route not only for managing RA symptoms, but also for maintaining a healthy BW and body composition [47]. Increased BW and body fat accumulation propels low-grade inflammation, thus, the adoption of the MD as an anti-inflammatory dietary pattern might induce a reduction in BW and tamper down inflammation. Herein, a 12-week MD intervention was effective at reducing BW, BMI, and FM among participants in the intervention arm compared with the controls.

RA, especially in the active disease state, is associated with considerable changes in blood lipids levels and insulin sensitivity, while metabolic changes, such as elevated TC, LDL, and TG concentrations, occur even in preclinical RA [63]. In the present study, TC, HDL, LDL, and TG remained unchanged in both study groups, but the TG levels were negatively associated with the MedDietScore, confirming the cardio-protective effects of the MD [24]. Additionally, the serum glucose levels were significantly lower among patients receiving personalized MD intervention compared with the controls, an observation that also implies the well-documented favorable effects of MD on glucoregulation [64]. 

There is evidence that the adoption of anti-inflammatory diets, including the MD, is negatively associated with inflammatory biomarkers in RA, such as CRP. In the randomized controlled study of Sköldstam and co-authors [53], patients with RA allocated to the MD arm exhibited significant reductions in CRP concentrations compared with the usual care group. Nevertheless, in the present study, CRP levels remained unchanged in both study arms, and this could be partially explained by the small sample size.

The role of vitamin D in immune-mediated inflammatory diseases, such as RA, has been extensively discussed in the literature. Vitamin D has been shown to exert immunomodulatory effects and downregulate pro-inflammatory agents [65]. Vitamin D deficiency is often observed in patients with RA [65,66], while hypovitaminosis is associated with the fastest disease progression, even in the early course of the disease [67]. A negative association between the serum vitamin D and RA disease activity has been reported, while vitamin D deficiency may be a potent contributor to CVD risk in RA [68]. In the present trial, 8 out of 40 patients with RA (20%) at the baseline had vitamin D insufficiency, with reference 1,25(OH)2D levels ranging between 20 to 68 ng/mL [69]. At the end of the trial, both study arms demonstrated a significant increase in the vitamin D serum levels, but the increment was sharper among participants in the intervention group than the control group at the end of the trial, with a mean concentration of 51.56 ng/mL vs. 40.36 ng/mL, respectively. The improvements noted in all study participants, irrespective of the treatment allocation, might be the result of the increased sunlight observed in the country during the summer months. Meta-analyses suggest that when vitamin D levels are improved (as in supplementation interventions), parallel improvements are also noted in the disease activity scores [66]. Thus, the ameliorated DAS28 observed at the end of the trial herein might well be the result of an overall improved vitamin D status.

Patients with RA tend to be inactive, in particular at the period following the diagnosis [70], with approximately 59% meeting the recommendations for PA [71]. In parallel, an RA diagnosis is reported as a persistent catalyst either for, or against, the performance of PA [72]. The use of nudges has been shown to increase PA levels and reduce sedentary behavior [73]. On the other hand, improvements in PA can also boost QoL and physical function [74], relieve pain, and increase aerobic capacity and CV fitness in RA [75,76]. In parallel, exercise is a known effector of circulating inflammatory markers [77] and an important contributor to cardiometabolic health [78]. In this manner, the improvements noted in the intervention arm herein might also be the result of greater PA among participants. In parallel, with improvements in PA and diet quality being parallel, it is also possible that they both consist of the result of nudging, or improved health-consciousness due to nudging.

Nudge policies aiming to modify health-related decisions, including dietary choices, are increasingly gaining popularity [79,80]. When nudge policies are delivered through CDSS platforms in particular, they can further improve the implementation of evidence [81]. According to a recent meta-analysis [82], a significant improvement in the proportion of patients receiving desired care is noted when CDSS are employed. Among patients with RA, previous studies have applied the CDSS for medication-related decisions [83] and for the incorporation of appropriate cultural contexts [84]. The present RCT consists of the first effort to use CDSS for improving lifestyle choices in RA.

Furthermore, the lifestyle intervention performed in the present RCT was carried out by experienced RDNs. Research has shown that when medical nutrition therapy (MNT) is applied by expert dietitians, patients tend to adhere to a greater degree and significant improvements are observed regarding their disease management [85,86,87,88]. In parallel, intensive lifestyle therapy, as performed herein, has also been shown to improve the health outcomes in comparison to standard care [85,89,90,91]. Furthermore, the use of CDSS for the delivery of interventions has been shown to improve clinical care [92]. Thus, it is possible that the additive effects of RDNs delivering the dietary intervention, the use of CDSS, and the more intensive meetings offered in the intervention arm may have corroborated for the observed improvements in the outcomes.

Patients with RA tend to underestimate the risk for developing CVD and are less concerned about the beneficial effects of protective lifestyle factors, such as the MD [93]. Therefore, personalized consultation sessions, as applied in the present trial, targeting improved MD adherence and higher levels of PA may have a beneficial impact not only with regard to the disease symptomatology, but also on the cardiometabolic and CV risk. There is evidence that increasing PA and/or exercise can simultaneously improve symptoms and reduce the impact of systemic RA manifestations [94]. A recent systematic review and meta-analysis revealed that the promotion of PA according to the public health recommendations improves CV fitness, muscle strength, and exercise behavior in patients with RA [76]. In the present trial, personalized lifestyle consultation sessions elevated the levels of PA of patients with RA at 12 weeks. Therefore, personalized nutrition and dietary guidance can promote diet quality and enhance disease management, serving as effective complementing treatment strategies for RA [95]. Such strategies can also enhance patient awareness on the value of lifestyle modification (healthy diet, regular exercise, smoking cessation, adequate sleep) on RA progression and remission. Based on the principles of the MD, Rondanelli and colleagues [96] have recently proposed a food pyramid adapted for patients with RA.

The small sample size and the possible bias that self-reported tools can cause comprise the limitations of the present study. For controlling these issues, a randomization protocol and validated questionnaires in Greek ample populations have been applied. In addition, the assigned RDNs were well experienced, and were able to detect unclear records and ask patients for proper clarifications.

## 5. Conclusions

In the present RCT, the delivery of a personalized diet plan based on the principles of the MD, paired with a lifestyle consultation for the promotion of PA for a total of 12 weeks, improved MD adherence in female patients with RA. Greater adherence to the MD was associated with an ameliorated dietary fat intake, BW, body composition, and lower disease activity state. Therefore, the adoption of the MD by patients with RA appears to be a feasible anti-inflammatory regime.

## Figures and Tables

**Table 1:** Inclusion and exclusion criteria for participation in the study.

Inclusion Criteria	Exclusion Criteria
(1) Adult women (≥ 18 years of age)	(1) Adolescent women
with an RA diagnosis according to the ACR/EULAR criteria [33] for >2 years	(2) Patients with altered treatment regime ≤ 6 months before, or during the trial
(2) With mild-moderate disease activity based on the DAS28 (DAS28 < 3.2)	(3) Women with RA with active disease (DAS28 > 5.1) [34]
	(4) People unable to read and comprehend the consent form
(3) On an unchanged treatment regime for >6 months	(5) Patients with psychiatric conditions
(4) Who provided consent for participation	(6) Pregnant or lactating women
	(7) Women on weight loss medication
	(8) Women following a vegan diet ≤ 5 years prior to screening
	(9) Those who did not consent or were unable to provide consent
	(10) Women with allergies, food intolerances, serious or life-threating illness, e.g., malignancy; infections; heart, liver, or renal failure; congenital metabolic diseases; malabsorption; or cognitive disorders
	(11) Alcoholism or drug addiction
	(12) Women taking vitamin or mineral supplementation during or ≤6 months prior to screening

ACR—American College of Rheumatology; DAS28—disease activity score 28 [34]; EULAR—European League Against Rheumatism; RA—rheumatoid arthritis.

**Table 2:** Characteristics of the participating women at baseline *.

Characteristics		Enrolled (N = 40)	Intervention Arm (*n* = 20)	Control Arm (*n* = 20)
	Age (years)	34.03 ± 5.45	34.15 ± 5.95	33.9 ± 5.06
Anthropometry:	BW (kg)	78.86 ± 19.46	79.19 ± 21.38	78.52 ± 17.88
	BMI (kg/m^2^)	25.76 ± 5.22	26.13 ± 5.89	25.40 ± 4.59
	FM (% of BW)	21.13 ± 12.07	20.63 ± 11.86	21.64 ± 12.56
	FFM (% of BW)	57.68 ± 11.92	57.53 ± 13.33	57.82 ± 10.66
Weight status:	Normoweight/Overweight/Obese (*n*)	22/14/4	12/6/2	10/8/2
Blood assays:	Glucose (mg/dL)	108.13 ± 17.16	110.61 ± 7.34	105.65 ± 23.19
	TC (mg/dL)	213.21 ± 50.11	209.22 ± 53.02	217.2 ± 48.07
	HDL (mg/dL)	69.33 ± 21.16	71.78 ± 17.62	66.88 ± 24.42
	LDL (mg/dL)	129.20 ± 42.31	130.02 ± 45.87	128.37 ± 39.61
	TG (mg/dL)	113.34 ± 67.12	112.23 ± 75.76	114.45 ± 59.20
	CRP (mg/dL)	0.74 ± 0.12	0.71 ± 0.11	0.77 ± 0.12
	1,25(OH)2D (ng/mL)	32.97 ± 4.10	33.79 ± 3.94	32.14 ± 4.19
Physical activity (IPAQ):	METs-min/week	647.46 ± 308.01	580.78 ± 322.39	714.14 ± 284.24
Adherence to the MD:	MedDietScore	38.25 ± 3.21	38.36 ± 3.46	38.14 ± 3.03
Daily dietary intake:	Total fat (g)	68.76 ± 9.49	67.02 ± 7.15	70.50 ± 11.29
	Dietary cholesterol (mg)	199.19 ± 45.49	188.94 ± 58.45	209.44 ± 24.70
	Fiber (g)	21.24 ± 4.85	19.98± 4.28	17.95 ± 4.24
	SFA (g)	18.98± 3.64	19.87 ± 2.16	18.09 ± 4.58
	MUFA (g)	29.63 ± 5.85	30.84 ± 5.47	28.42 ± 6.11
Disease activity:	DAS28	2.82 ± 0.19	2.83 ± 0.19	2.81 ± 0.20

1,25(OH)2D—1,25-Dihydroxyvitamin D; BW—body weight; BMI—body mass index; CRP—C-reactive protein; DAS28—disease activity score 28 joints [34]; FFM—fat-free mass; FM—fat mass; HDL—high-density lipoprotein; IPAQ—International Physical Activity Questionnaire [37]; LDL—low-density lipoprotein; MD—Mediterranean diet; MedDietScore—Mediterranean Diet Score [36]; MET—metabolic equivalents of task; MUFA—mono-unsaturated fatty acids; NS—not significant; SFA—saturated fatty acids; TC—total cholesterol; TG—triglycerides; * Data are expressed as counts (*n*) or mean values ± standard deviation of mean (SD).

**Table 3:** Characteristics of the participating women at baseline and end of treatment (12 weeks) *.

Characteristics		Intervention Arm (*n* = 20)		*p*-Values within Timepoints ^†^	Control Arm (*n* = 20)		*p*-Values within Timepoints ^†^	*p*-Values between Groups ^†^		*p*-Values between Groups ^†^
		before	after		before	after		Baseline	12 Weeks	Δ
Anthropometry:	BW (kg)	79.2 ± 21.4	77.1 ± 20.8	<0.001	78.5 ± 17.9	80.6 ± 18.3	<0.001	0.916	0.576	<0.001
	BMI (kg/m^2^)	26.13 ± 5.89	25.42 ± 5.63	<0.001	25.40 ± 4.59	26.04 ± 4.58	<0.001	0.663	0.702	<0.001
	FM (% of BW)	20.6 ± 11.9	20.3 ± 12.1	<0.001	21.6 ± 12.6	22.1 ± 12.1	0.002	0.794	0.665	<0.001
	FFM (% of BW)	57.5 ± 13.3	56.7 ± 13.3	0.001	57.8 ± 10.7	57.8 ± 11.1	0.954	0.940	0.774	0.348
Blood assays:	Glucose (mg/dL)	110.6 ± 7.3	104.8 ± 5.3	<0.001	105.7 ± 23.2	104.7 ± 23.2	0.401	0.368	0.964	0.005
	TC (mg/dL)	209.2 ± 53.0	200.6 ± 56.5	0.183	217.2 ± 48.1	228.8 ± 53.0	0.209	0.621	0.111	0.071
	HDL (mg/dL)	71.8 ± 17.6	69.7 ± 19.2	0.680	66.9 ± 24.4	73.4 ± 27.3	0.133	0.472	0.625	0.193
	LDL (mg/dL)	130.0 ± 45.9	126.5 ± 52.0	0.637	128.4 ± 39.6	132.3 ± 45.3	0.623	0.904	0.708	0.493
	TG (mg/dL)	112.2 ± 75.8	97.5 ± 57.7	0.057	114.5 ± 59.2	126.3 ± 83.6	0.380	0.918	0.212	0.086
	CRP (mg/dL)	0.71 ± 0.11	0.72 ± 0.11	0.408	0.77 ± 0.12	0.75 ± 0.11	0.367	0.084	0.460	0.240
	1,25(OH)_2_D (ng/mL)	33.8 ± 3.9	51.6 ± 11.8	<0.001	32.1 ± 4.2	40.4 ± 6.4	<0.001	0.208	<0.001	0.001
Physical activity (IPAQ):	METs (min/week)	580.8 ± 322.4	616.5 ± 315.9	0.002	714.1 ± 284.2	684.7 ± 251.1	0.065	0.174	0.454	0.002
MD adherence:	MedDietScore	38.36 ± 3.46	42.11 ± 2.48	<0.001	38.14 ± 3.03	38.36 ± 2.74	0.359	0.829	<0.001	<0.001
Daily dietary intake:	Total fat (g)	67.02 ± 7.15	61.19 ± 7.90	<0.001	70.50 ± 11.29	73.42 ± 11.57	<0.001	0.251	<0.001	<0.001
	Cholesterol (mg)	188.9 ± 58.5	169.2 ± 50.3	<0.001	209.4 ± 24.7	224.6 ± 28.3	<0.001	0.157	<0.001	<0.001
	Fiber (g)	19.98 ± 4.28	26.09 ± 5.00	<0.001	17.95 ± 4.24	23.73 ± 4.24	0.067	0.140	0.116	<0.001
	SFA (g)	19.87 ± 2.16	16.73 ± 3.12	0.004	18.09 ± 4.58	18.48 ± 4.77	0.132	0.125	0.177	0.001
	MUFA (g)	30.84 ± 5.47	36.98 ± 5.82	<0.001	28.42 ± 6.11	28.73 ± 5.38	0.433	0.195	<0.001	<0.001
Disease activity:	DAS28	2.83 ± 0.19	2.71 ± 0.14	<0.001	2.81 ± 0.20	2.80 ± 0.17	0.804	0.744	0.054	<0.001

1,25(OH)2D—1,25-Dihydroxyvitamin D; Δ—change from baseline to end of treatment; BW—body weight; BMI—body mass index; CRP—C-reactive protein; DAS28—disease activity score 28 joints [34]; FFM—fat-free mass; FM—fat mass; HDL—high-density lipoprotein; IPAQ—International Physical Activity Questionnaire [37]; LDL—low-density lipoprotein; MD—Mediterranean diet; MedDietScore—Mediterranean Diet Score [36]; MET—metabolic equivalents of task; MUFA—mono-unsaturated fatty acids; NS—not significant; SFA—saturated fatty acids; TC—total cholesterol; TG—triglycerides. * Data are expressed as mean values ± standard deviation of mean (SD); ^†^ *p*-value: significant differences between the control and the intervention group at baseline analyzed by independent sample *t*-test or the Mann–Whitney U test, where applicable.

## Data Availability

All data are available upon request to the first author.
